# Blended Teaching Methodology of E-learning and Simulation Training in Obstetrics and Gynecology for Undergraduate Medical and Nursing Trainees

**DOI:** 10.7759/cureus.40062

**Published:** 2023-06-06

**Authors:** K Aparna Sharma, L Levis Murry, Juhi Bharti, Ravneet Kaur, Jyoti Meena, Vidushi Kulshrestha, Sadia Mansoor, Snigdha Soni, Sunesh Kumar

**Affiliations:** 1 Obstetrics and Gynaecology, All India Institute of Medical Sciences, New Delhi, IND; 2 College of Nursing, All India Institute of Medical Sciences, New Delhi, IND; 3 Centre for Community Medicine, All India Institute of Medical Sciences, New Delhi, IND; 4 Gynaecological Oncology, All India Institute of Medical Sciences, New Delhi, IND

**Keywords:** blended learning, gynaecology and obstetrics, nursing education, medical education, undergraduate teaching, simulation

## Abstract

Context

The concept of simulation-based teaching has become a standard practice for health education in the present era. However, there is a paucity of literature on integrating simulation-based teaching in the conventional training of undergraduate medical and nursing students.

Aim

To explore the effectiveness and benefits of e-learning along with low-fidelity simulation in obstetrics and gynecology among undergraduate medical and nursing students at a tertiary care center in India.

Methodology

It was a prospective study conducted on 53 final-year undergraduate medical students and 61 final-year undergraduate nursing students. All students underwent a knowledge-based pre-test followed by exposure to an e-learning module on four selected obstetrics and gynecology skills, namely, conducting normal delivery, episiotomy suturing, pelvic examination, and insertion of intrauterine device. Students practiced these four skills on low-fidelity simulators. After this, they underwent a post-test assessment and gave feedback. A focused group discussion was conducted to explore their experiences.

Results

There was a statistically significant difference between the pre-test and post-test knowledge scores of all the students (p =< 0.001). Students found this teaching strategy useful and reported an increase in self-assessed confidence. Focused group discussion revealed various themes like improved satisfaction and ability to practice repeatedly without fear of harming patients.

Conclusions

Based on the results, this teaching methodology should be integrated as an adjunct method of teaching in the undergraduate curriculum from the first year itself, which will motivate students to participate in clinical care and will result in quality improvement of health care.

## Introduction

A critical learning outcome of any healthcare education program is to produce students who are competent to provide safe and effective clinical care. With the advent of newer technologies, it has been possible to use online teaching, simulation-based education, and blended learning in health care. Simulation in medical education can be defined as replicating a clinical scenario using mannequins or human actors to make the learning process of a skill or procedure easier and understandable [1]. Simulation-based education is related to improved confidence, better attention to human factors in learning, self-efficacy, and improved team performance in students [2-4].

Simulation-based education has expanded globally in different specialties of health care, including obstetrics [5,6], anesthesia [7], emergency medicine [8], and cardiology [9]. Medium to low fidelity simulation has also been used to train nurses in India [10-12], with reported effectiveness. As far as the obstetrics and gynecology field is concerned, topics like pelvic examination and normal delivery pose a practical challenge to educators. An increasing number of trainees and a declining number of basic skills per student has reduced learning opportunities in the present scenario [13].

In our setup, undergraduate medical and nursing students are expected to learn and perform basic skills in obstetrics and gynecology like conducting a normal delivery, suturing episiotomy, carrying out the pelvic gynecological examination, and insertion of copper T. They are exposed to these procedures during their clinical postings but often, the patients do not give consent for repeated examinations or teaching at the same time. Therefore, these students have limited opportunities to learn the skills and are underconfident. Simulation is an exciting alternative that allows them to practice these skills on mannequins for preliminary learning as many times as they wish. Thus, it helps in bridging the gap between theoretical knowledge and practice in real-life situations.

Challenges such as lack of structural training, low skill level, resistance to change among students, and nurse-nurse hierarchy have been reported in the implementation of simulation for nurses [14]. The use of simulation in healthcare education in India is in its incipient stage and there is a dearth of reported studies relating to the use of this teaching method. A bigger challenge is also to integrate simulation-based teaching into the conventional curriculum. Due to the paucity of literature on integrating simulation into the conventional training of undergraduate students, we conducted this study to explore the feasibility and effectiveness of simulation-based learning in undergraduate medical and nursing students at our institute.

## Materials and methods

This study was conducted in SET (skill lab, e-learning, & telemedicine) facility, All India Institute of Medical Sciences, New Delhi. The Blended e-Learning and Simulation-based Training (Be-ST) modules were developed by the faculty of obstetrics and gynecology (OBG), who are trained in skills training, e-learning, and telemedicine at the institute. Each module has online content with recorded lectures, containing standard operating procedures (SOPs), videos demonstrating skills, and multiple choice questions related to the module. The developed online content was shared by email to all the Faculty of OBG as well as to the faculty of other disciplines trained in skills, e-learning, and telemedicine at the institute for peer review. The suggestions were incorporated, and revised content was shared for standardization and validation by these neutral observers as well as other student groups.

The four modules included in the present study were the conduct of normal delivery, episiotomy repair, gynecological pelvic examination, and copper T insertion. The study was conducted on 53 final-year undergraduate medical students and 61 final-year undergraduate nursing students during their clinical posting in the department of OBG. The training was conducted throughout the year targeting the whole batch of undergraduates interspersed at the times when they were posted in the clinical department providing accessibility to facilitators and flexibility of learning. Prior ethical approval was taken from the institute's ethics committee for the study. Informed written consent was taken from all the participants.

Before the access to online content, all the students underwent a pre-test knowledge assessment through 20 multiple choice questions, with five questions per module (Appendix 1). After this, they were provided access to the online content through Online Neonatal Training and Orientation Programme (ONTOP) platform. At the completion of the online content, a certificate was generated, which was a prerequisite for attending the practical simulation session.

After this, the students underwent hands-on training in the skills lab on mannequins under the supervision of the faculty. The students were divided into groups with six to seven members each, spending around 45 minutes to one hour at each station. After the hands-on training, a post-test knowledge assessment was done using the same questions (Appendix 1) and compared with the pre-test score. A structured feedback form based on a five-point Likert scale was filled by all the students at the end of the session. It consisted of 10 questions. Qualitative assessment was done by focused group discussion (FGD) at the end of the session for four groups (two each of nursing and medical students) consisting of eight students each to assess their experience of the new teaching methodology.

For statistical analysis, data were entered into a Microsoft Excel spreadsheet (Microsoft Corporation, Redmond, WA), and then imported to STATA software version 13.1 (Stata Corp, College Station, TX). Descriptive statistics, such as mean, standard deviations, and range values, were obtained. Comparison of score changes between pre and post were tested using Student's t-paired test. The level of significance was taken to be <0.05. The data from the FGDs were transcribed verbatim, and data were coded into categories.

## Results

All of the students were exposed to the e-learning module in OBG for the first time. Statistical difference was found between the pre-test and post-test knowledge scores of both the medical and nursing students (Table 1). However, this difference was quite high among the nursing students. The summary of feedback by students is given in Table 2. The majority of them (94.1% medical vs. 70.9% nursing students) opined that the stations selected were extremely relevant to their clinical practice with an average rating of 4.9 and 4.5 out of 5, respectively. Almost all of them reported an increase in confidence to perform the skills in actual clinical settings after their hands-on training. Remarkably, 100% of the medical students thought that the lectures should be completely replaced by a hybrid mode of teaching with e-learning supported by simulation-based training and more such skills should be included in the undergraduate curriculum. The nursing students also clearly preferred this method of teaching over lectures (rating 4 = 41.8% and rating 5 = 54.5%). The difference in mean scores of pre and post was highly significant (p < 0.001), which resulted in more than 90% power post-hoc test.

**Table 1:** Pre- and post-intervention knowledge scores of study participants

Category	Pre-test score, Mean (SD)	Post-test score, Mean (SD)	P-value
Medical students (n = 53)	17.2 (2.0)	18.7 (1.3)	<0.001
Nursing students (n = 61)	8.0 (2.3)	16.8 (1.4)	<0.001
Overall (n = 114)	12.3 (5.1)	17.7 (1.6)	<0.001

**Table 2:** Feedback from undergraduate medical and nursing students based on a five-point Likert scale SOPs: standard operating procedures; MCQs: multiple choice questions.

S. No.	Variable	Average rating (SD) (Medical, n = 53)	Average rating (SD) (Nursing, n = 55)
1	How relevant are the stations for your clinical practice? (1 = Not important, 5 = Extremely important)	4.9 (0.27)	4.5 (0.83)
2	What is the overall benefit of the skill module? (1 = No benefit, 5 = Very useful)	4.7 (0.57)	4.3 (0.79)
3	How would you grade the online content? (1 = Not good, 5 = Excellent); SOPs, videos, MCQs	4.4 (0.60), 4.0 (0.84), 4.4 (0.68)	4.0 (0.97), 3.9 (1.1), 3.7 (1.18)
4	How would you rate the hands-on training? (1 = Not good, 5 = Excellent)	4.7 (0.46)	4.0 (0.79)
5	How confident were you in performing these skills before this module? (1 = Not at all confident, 5 = Very confident)	2.3 (1.14)	2.2 (1.22)
6	How confident do you feel to perform these skills in actual clinical settings after hands-on training? (1 = Not at all confident, 5 = Very confident)	4.0 (0.60)	3.8 (0.69)
7	How would you rate this teaching session when compared to lectures? (1 = Very low, 5 = Very high)	5.0 (0)	4.5 (0.63)
8	Has this session improved your understanding of the subject when compared to your last posting in gynecology in the third year? (1 = No difference, 5 = Significant difference)	4.6 (0.68)	4.4 (0.85)
9	Would you recommend this simulation-based education to your colleagues? (1 = Not at all, 5 = Definitely recommend)	4.9 (0.27)	4.8 (0.50)
10	Would you suggest more similar stations to be included in your curriculum in the future? (1 = Strongly disagree, 5 = Strongly agree)	4.9 (0.34)	4.8 (0.69)

From the FGD, it was obvious that the students liked this new teaching methodology and rated this way better than traditional classroom teaching. The main highlights by medical (M) and nursing (N) students are provided below.

How was your experience of learning clinical procedures in the skill lab?

Overall, all the students were more satisfied with this teaching methodology. It boosted their confidence and made the subject more interesting than ever before.

They stated the following: it was nice and much better than classes as learning is better by doing things rather than listening in classrooms (M). Great experience! Very happy (N). Lectures should be replaced by newer teaching methodologies like simulation-based teaching, and group discussions (M). It boosts confidence before doing it on patients (both). Learning is better, as we can do it multiple times till we are satisfied (M).

What are the advantages of learning a skill in a skill lab as compared to real patients?

The main advantages of the simulation were that the stations were very interactive, and they were free to do it repeatedly till they understood properly.

They said the following: there is an option to ask doubts in the process of learning, unlike a lecture, which is often one-sided (M). This method is better for our preliminary learning… later can practice and get better on patients… should be implemented for all the procedures that we have to learn (M). It is awkward to ask questions from faculty and residents in the outpatient department and delivery room where the patient is already in distress (both). We can make mistakes and correct them at the same time without any fear (N). A better understanding of the skills helps in answering the exam (M).

What are the disadvantages of learning a skill in a skill lab as compared to real patients?

The limitations of this teaching methodology were that there was a lack of realism and mannequins were low fidelity with problems of wear and tear due to repeated usage.

They stated the following: the feel is never going to be as accurate as in a real patient (M). Dummies were not that realistic... not close to real life (N). Limited time to practice (N).

What are the factors that motivated you to attend this skill-based program?

The students had never attended any simulation-based teaching in OBG in the past. The reasons for good attendance were: it was conveyed that attendance is mandatory (M). The previous Neonatal Resuscitation Protocol session at the pediatrics department was good (M). We wanted to do it before the real exposure (N).

What is your feedback on online content?

Though the students liked the SOPs, they found them monotonous and repetitive. They opined: when we are taught the procedure in clinics, there is something different and left out… few steps are forgotten or not mentioned or thought irrelevant in the clinics... but SOPs make it standard and complete (M). Text is not attractive, is repeated in videos, and should have more diagrams and illustrations (M).

Would you recommend any more skills to be included in the curriculum?

Students felt that all possible skills should be taught by simulation in skill labs, especially the ones they are required to perform during internship and village posting. They wanted to have similar modules in other specialties too and suggested to have them start from the basic procedures like intravenous cannulation and injections.

Suggestions to improve

Students suggested that mannequins could be of better quality and high fidelity to mimic real-life situations more closely. They strongly felt that such type of teaching should be started from the fourth semester itself and they should have access to website and online content as and when they wished with no time restrictions.

## Discussion

This study was conducted to assess the feasibility and effectiveness of the Blended e-Learning and Simulation-based Training (Be-ST) modules on final-year undergraduate medical and nursing students. There was a statistically significant improvement in the post-test knowledge scores of the students as compared to their pre-test scores in both groups. This may be attributed to the web-based learning modules, which were further reinforced by task training. It can be noted that the difference in scores was markedly higher in nursing students as compared to medical students. It may be due to differences in the structure of teaching in both the groups where medical students at our institute are exposed to lectures and clinical postings in OBG from the fourth semester itself.

The core component of the undergraduate curriculum is learning clinical skills, which has dropped markedly due to multiple reasons. In a retrospective study [13], authors have reported significant changes in the trend of OBG cases in the past decade in the United States. They have documented a decline in the number of vaginal deliveries and a reduction in operative vaginal deliveries and an increase in the cesarean sections per resident. Therefore, innovative methods like simulation help in bridging the gap between theory and practice and is of utmost importance in learning basic clinical skills in the present era.

The students strongly felt that newer teaching methodologies should replace conventional classroom lectures. A recent randomized trial [15] compared the efficacy of two teaching strategies on skill learning in medical students during internship and concluded that training is better if done on mannequins prior to patients. Similar experiences had been reported by first-year nursing students who had undergone high-fidelity simulation learning in Japan [16]. Well-designed simulation exercises [17] have the potential to prevent medical errors. The importance of simulation to prevent medical errors and patient harm can be inculcated among the students right from their training by introducing simulation in the curriculum.

The students also reported increased confidence in performing the skills after undergoing the module. In a study [5] designed to assess the role of simulation in vaginal delivery during undergraduate medical training in OBG, participation in live deliveries was significantly more in the simulated group as compared to the non-simulated group. Students of the simulated group reported an increase in self-assessed confidence in many aspects of conducting normal vaginal deliveries. The present study also has similar findings of an increase in self-assessed confidence after hands-on training among 82% of medical students. When asked about the confidence to perform the skills in actual clinical settings, the average rating was 2.3 before the hands-on training, which rose to 4 out of 5 after hands-on training. Similarly, the effectiveness of simulation in increasing knowledge and confidence in nursing students also has been reported in other studies [18-20]. A survey [21] of final-year nursing students in India reported that hands-on skill practice and supervised clinical practice were associated with high confidence in basic midwifery skills. In a recent study from India, the authors introduced an interprofessional simulation-based program (WHIPLS-Women's Health Interprofessional Learning by Simulation) for undergraduate medical and midwifery teaching in obstetrics and gynecology. Students reported an increase in confidence in performing the skills. Both the medical and midwifery students rated the overall benefit of the workshop as 4.6 and 4.3, respectively, out of five [22].

A Canadian study [23] was conducted to assess the role of simulation in improving the skills for managing postpartum hemorrhage among third-year medical undergraduates. Here, high-fidelity simulation was used, and it showed a significant improvement in post-test scores by 13% (p < 0.01). The majority of them (93%) enjoyed the new teaching methodology over conventional methods [22]. Similar findings were there in the present study where 100% of medical students and 96% of nursing students preferred simulation-based teaching and other new methods that make the subject interesting, enjoyable, and more beneficial.

A key learning from the study has been the integration of simulation-based teaching into the conventional curriculum using the Be-ST modules. Using this methodology, we were able to establish the preliminary content for teaching, the target audience to focus on, the positioning of the modules in the undergraduate curriculum, and the method of content delivery. This will be vital learning for the future as this will help in building the base for integrating other modules to be taught at various other levels for undergraduate and postgraduate teaching and this is a proof of concept for the implementation of the skill component of the competency-based curriculum that is being adopted across various countries in the world.

The limitations of the study have been logistic issues related to a lack of realism owing to low-fidelity mannequins. In this study, the intermediate and long-term impact on learning and retaining knowledge and skills were not assessed. It would be interesting to see the effect in actual clinical settings. Another limitation is the lack of a control group for comparison of training by web-based module alone versus simulation-based module, to further add which mode is the most influential. Further studies are required with larger sample sizes and smaller groups to address these concerns.

## Conclusions

Despite the limitations, students reported improvement in skills and self-assessed confidence, clarity in concepts, reduction in fear of making mistakes, and a desire to participate in such teaching sessions in each year of their professional training. This teaching methodology should be integrated as an adjunct method of teaching in the undergraduate curriculum. This will facilitate greater participation of students in clinical care during undergraduate training and internship with an impact not only on the overall performance in exams but also on healthcare delivery. Simulation-based education is the need of the hour for undergraduate teaching and it gives a positive clinical experience to the students.

## Appendices

Appendix 1: Multiple choice questions (pre- & post-test questionnaire)

Q1: All the following instruments are required for pelvic examination except:

1. Sim’s speculum

2. Cusco’s speculum

3. Anterior vaginal wall retractor

4. Uterine sound

Q2: All the following are pre-requisites of pelvic examination except:

1. Verbal consent

2. Illumination

3. Full bladder

4. Female chaperone should be available.

Q3: Which of the following statement is false regarding pelvic examination?

1. It is a basic skill required in gynecological OPD

2. Bimanual examination is done prior to speculum examination

3. Cusco’s speculum is self-retaining

4. It is done in a dorsal position

Q4: All the following describe the findings of a normal uterus except:

1. Mobile

2. Pear shaped

3. Tender

4. Firm

Q5. What do you mean by bimanual pelvic examination?

1. Lower abdominal palpation by both hands

2. Pelvic examination using one finger of both hands vaginally

3. Pelvic and per rectal examination together

4. Palpation of pelvic organs by per vaginal examination using two fingers of one hand along with the other hand in hypogastrium

Q6: What kind of perineal tear is an episiotomy?

1. First degree

2. Second degree

3. Third degree

4. Fourth degree

Q7: Episiotomy is usually indicated in all the following except:

1. Forceps delivery

2. Multigravida

3. Rigid perineum

4. Breech delivery

Q8: Which of the following muscle is not severed in episiotomy?

1. Bulbospongiosus

2. Ischiocavernosus

3. Superficial transverse perinei

4. Deep transverse perinei

Q9: Which suture is used in episiotomy repair?

1. Silk 1-0

2. Polyglactin 910 2-0

3. Prolene 2-0

4. Nylon 2-0

Q10. What is the best time for giving an episiotomy?

1. In between two contractions when the patient is relaxed

2. As soon as the head can be seen at the vulva/perineum

3. At the height of contractions at the time of crowning

4. After the delivery of the anterior shoulder

Q11. Under the National Family Welfare Programme, the currently available Cu-T 380 A is effective for a period of

1. Three years

2. Five years

3. Eight years

4. Ten years

Q12. Interval Cu-T insertion means insertion at any of the following times EXCEPT

1. Any time during the menstrual period, after pregnancy is ruled out

2. After six weeks of delivery

3. Within four days of unprotected sexual intercourse

4. After 12 days of complete abortion

Q13. Which of the following is NOT a warning sign for a Cu-T user?

1. Pain during sexual intercourse

2. Crampy lower abdominal pain

3. Not able to feel the threads

4. Heavier or longer bleeding during the first few weeks of Cu-T insertion

Q14. Which of the following statements is true regarding Cu-T insertion?

1. No anesthesia is required for Cu-T insertion

2. Cu-T insertion can be done even if pregnancy status cannot be confirmed

3. Routine laboratory investigations should be done before Cu-T insertion

4. A woman should use some alternate method of contraception for the first seven days of Cu-T insertion

Q15. Cu-T can be safely used in all of the following conditions except:

1. Lactating women

2. Unexplained vaginal bleeding

3. Women taking anti-hypertensive drugs

4. Women having anemia

Q16. Which is the correct sequence of cardinal movements?

1. Descent-flexion-restitution-extension-internal rotation-external rotation-delivery

2. Descent-flexion-internal rotation-extension-restitution-external rotation-delivery

3. Descent-flexion-internal rotation-restitution-external rotation-extension-delivery

4. Descent-flexion-extension-internal rotation-restitution-external rotation-delivery

Q17. Crowning is best defined as:

1. When the greatest diameter of the fetal head comes through the vulva

2. When the fetal head is at the introitus and does not recede in between contractions

3. When the head is delivered

4. When presenting part reaches the pelvic floor

Q18. Dose of oxytocin in active management of the third stage of labor (AMTSL):

1. Injection oxytocin 10 U IV

2. Injection oxytocin 5 U IV

3. Injection oxytocin 10 U intramuscular

4. Injection oxytocin 5 U IV

Q19. Desirable site of episiotomy

1. Lateral episiotomy

2. J-shaped episiotomy

3. Median episiotomy

4. Right mediolateral episiotomy

Q20. Active management of the third stage includes all except?

1. Uterine massage

2. Suprapubic massage

3. Injection of oxytocin after delivery

4. Controlled cord traction

## References

[1] Maran NJ, Glavin RJ (2003). Low- to high-fidelity simulation - a continuum of medical education?. Med Educ.

[2] So HY, Chen PP, Wong GK, Chan TT (2019). Simulation in medical education. J R Coll Physicians Edinb.

[3] Shin S, Park JH, Kim JH (2015). Effectiveness of patient simulation in nursing education: meta-analysis. Nurse Educ Today.

[4] Franklin AE, Lee CS (2014). Effectiveness of simulation for improvement in self-efficacy among novice nurses: a meta-analysis. J Nurs Educ.

[5] Dayal AK, Fisher N, Magrane D, Goffman D, Bernstein PS, Katz NT (2009). Simulation training improves medical students' learning experiences when performing real vaginal deliveries. Simul Healthc.

[6] Scholz C, Mann C, Kopp V, Kost B, Kainer F, Fischer MR (2012). High-fidelity simulation increases obstetric self-assurance and skills in undergraduate medical students. J Perinat Med.

[7] Morgan PJ, Cleave-Hogg D, McIlroy J, Devitt JH (2002). Simulation technology: a comparison of experiential and visual learning for undergraduate medical students. Anesthesiology.

[8] Smolle J, Prause G, Smolle-Jüttner FM (2007). Emergency treatment of chest trauma--an e-learning simulation model for undergraduate medical students. Eur J Cardiothorac Surg.

[9] Morgan PJ, Cleave-Hogg D, Desousa S, Lam-McCulloch J (2006). Applying theory to practice in undergraduate education using high fidelity simulation. Med Teach.

[10] Faucher MA, Riley C, Prater L, Reddy MP (2016). Midwives in India: a delayed cord clamping intervention using simulation. Int Nurs Rev.

[11] Ghosh R, Spindler H, Morgan MC (2019). Diagnosis and management of postpartum hemorrhage and intrapartum asphyxia in a quality improvement initiative using nurse-mentoring and simulation in Bihar, India. PLoS One.

[12] Vail B, Morgan MC, Spindler H, Christmas A, Cohen SR, Walker DM (2018). The power of practice: simulation training improving the quality of neonatal resuscitation skills in Bihar, India. BMC Pediatr.

[13] Gupta N, Dragovic K, Trester R, Blankstein J (2015). The changing scenario of obstetrics and gynecology residency training. J Grad Med Educ.

[14] Morgan MC, Dyer J, Abril A, Christmas A, Mahapatra T, Das A, Walker DM (2018). Barriers and facilitators to the provision of optimal obstetric and neonatal emergency care and to the implementation of simulation-enhanced mentorship in primary care facilities in Bihar, India: a qualitative study. BMC Pregnancy Childbirth.

[15] Bhutta SZ, Yasmin H (2018). Comparative effectiveness of teaching obstetrics and gynaecological procedural skills on patients versus models: a randomized trial. Pak J Med Sci.

[16] Lee SJ, Kim SS, Park YM (2015). First experiences of high-fidelity simulation training in junior nursing students in Korea. Jpn J Nurs Sci.

[17] Sarfati L, Ranchon F, Vantard N (2019). Human-simulation-based learning to prevent medication error: a systematic review. J Eval Clin Pract.

[18] Tubaishat A, Tawalbeh LI (2015). Effect of cardiac arrhythmia simulation on nursing students’ knowledge acquisition and retention. West J Nurs Res.

[19] Eyikara E, Baykara ZG (2018). Effect of simulation on the ability of first year nursing students to learn vital signs. Nurse Educ Today.

[20] Tamaki T, Inumaru A, Yokoi Y (2019). The effectiveness of end-of-life care simulation in undergraduate nursing education: a randomized controlled trial. Nurse Educ Today.

[21] Sharma B, Hildingsson I, Christensson K (2019). The association of teaching-learning methods and self-confidence of nurse-midwives. A survey from one province in India. Women Birth.

[22] Gorantla S, Bansal U, Singh JV, Dwivedi AD, Malhotra A, Kumar A (2019). Introduction of an undergraduate interprofessional simulation based skills training program in obstetrics and gynaecology in India. Adv Simul (Lond).

[23] Shore EM, Davidson A, Arnason M, Kara H, Shah A, Shah R (2019). Bridging the gap: incorporating simulation into obstetrics and gynaecology undergraduate medical education. J Obstet Gynaecol Can.

